# Limitations in the implementation of control measures for bovine paratuberculosis in infected Swiss dairy and beef herds

**DOI:** 10.1371/journal.pone.0245836

**Published:** 2021-02-02

**Authors:** Myriam Klopfstein, Alexandra Leyer, Beat Berchtold, Paul Robert Torgerson, Mireille Meylan

**Affiliations:** 1 Clinic for Ruminants, Vetsuisse Faculty, University of Bern, Bern, Switzerland; 2 Institute for Food Safety and Hygiene, Section of Veterinary Bacteriology, Vetsuisse Faculty, University of Zurich, Zurich, Switzerland; 3 Section of Veterinary Epidemiology, Vetsuisse Faculty, University of Zurich, Zurich, Switzerland; National Veterinary School of Toulouse, FRANCE

## Abstract

Various measures have been advocated for the control of Johne’s disease (caused by *Mycobacterium avium* subsp. *paratuberculosis*, MAP) in different countries. Farmers’ compliance has been reported to be variable depending on disease prevalence and incentives to participate in control programs. After the prevalence of MAP shedding and risk factors for within-herd spread of MAP were assessed in 17 Swiss cattle herds (10 dairy and 7 beef), general and herd-specific recommendations were given to the farmers to reduce MAP transmission within the herd. Participation in the study and implementation of control measures were voluntary, no financial incentives were provided for the realization of control measures. After a 3-year period of monitored observation including biannual farm visits and discussion of the situation, the implementation of the recommended control measures and their effect on prevalence of MAP shedding were evaluated. Implementation of recommended general and farm-specific control measures was only partially realized. Neither the number of animals tested positive (before or during the study) nor the farmers’ knowledge about paratuberculosis were significantly associated with their compliance for the implementation of management changes. The apparent within-herd prevalence remained constant despite limited implementation of control measures, and no particular group of control measures was found to be associated with changes in prevalence. Farmers’ compliance for the implementation of control measures to reduce the impact of Johne’s disease in infected farms was very limited under Swiss farming conditions in the frame of voluntary participation in a research project. These results indicate that the losses associated with paratuberculosis in Swiss dairy and beef operations are not estimated to be high enough by the farmers to justify important efforts for control measures, and that incentives may be necessary to achieve efficient implementation of such measures.

## Introduction

Paratuberculosis in cattle, caused by infection with *Mycobacterium avium* subsp. *paratuberculosis* (MAP), is associated with chronic profuse diarrhea, weight loss and decreased milk yield, and therefore leads to economic losses in affected farms worldwide [1–3]. As an effective treatment for bovine paratuberculosis (PTB) does not exist, control measures should focus on preventive strategies to keep non-infected herds disease-free and to reduce the rate of new infections within infected herds [4].

The agricultural structure of a country affects the frequency of contact between cattle from different herds and thus influences the spread of Johne’s disease (JD). Animal purchase is common in Switzerland, and young stock from different farms are often raised together in specialized heifer-rearing farms or come together during summer season on alpine pastures. As in other countries, animal purchase has been shown to be the most important risk factor associated with an infected herd status in Switzerland [5–8].

Control programs for PTB aiming at reducing the spread of MAP within infected herds, including test-and-cull strategies and measures to improve management practices, have been conducted in different countries [9, 10]. Conceptual design and success of control programs to minimize new infections in MAP positive herds depend on agricultural structures such as herd size, husbandry and biosecurity practices, as well as on the prevalence of infection in the herds. Furthermore, various infection pathways are described for PTB and recommendations to reduce MAP transmission are accordingly numerous. The identification of essential management practices to prevent new infections in a given situation is therefore a crucial tool for the success of a control program [11]. Under Swiss farming conditions, contamination of the heifer area with manure from adult cattle and management practices favoring the exposure of newborn animals to feces of adult animals in the calving area were found to be the main risk factors associated with a high within-herd prevalence [6].

Beside the assessment of factors influencing MAP spread within infected herds, adequate information of the participating farmers and financial incentives can influence the success of a control program [12, 13]. In a previous Swiss study, the knowledge level of the managers of infected dairy and beef herds about PTB was limited, and it was very low in control herds as 40% of the managers of uninfected dairy herds and 32% of the managers of uninfected beef herds had never heard of the disease [6].

After assessing the apparent prevalence of MAP shedding and the risk factors potentially associated with MAP transmission in 17 Swiss dairy and beef farms in a previous study [6], general and individual recommendations to control further spread of MAP within the herd were given for each farm. The main objective of the present study was to assess the farmers’ compliance regarding the implementation of recommended (non-mandatory) control measures in the absence of financial incentives under Swiss farming conditions during a three-year period of observation (PO). Within-herd prevalence of MAP shedding was assessed at the start and at the end of the PO.

## Materials and methods

### Farm visits, recommended control measures and data collection

Seventeen MAP infected farms were included in the study, ten dairy herds (herds A-J) and seven beef herds (herds K-Q). Participation to the study was voluntary. The farms were recruited and visited for the first time between February and July 2011. Husbandry practices were assessed in detail by use of two specific questionnaires, one for dairy and one for beef herds, and the prevalence of MAP shedding was established by use of fecal cultures (first testing). Additional information about PTB cases on the farm in the past and the farmers’ knowledge about the disease was collected at that time as well [6].

At the end of the first farm visit, the most important measures applied in other countries in the frame of PTB control programs [4, 9–11, 14–19] to limit within-herd MAP transmission in dairy and beef operations were discussed with the herd managers (Table 1).

**Table 1:** List of the general recommendations given to the managers of ten dairy and seven beef farms to limit MAP spread on their farm.

Recommended general control measures	Dairy herds	Beef herds
Culling of test-positive animals and their offspring	x	x
Avoiding / minimizing animal purchase	x	x
Good hygiene during calving	x	x
No feeding of infected colostrum of milk	x	N/A
Avoiding contact between calves and cows	x	N/A
Avoiding contact between heifers and cows	x	x
Avoiding contamination of the calf area with cow manure	x	N/A
Avoiding contamination of feed or water	x	x
Good hygiene in the farm	x	x
Pasture management to avoid exposure of calves to MAP	x	x
Fencing of manure stock and pit	x	x

MAP: *Mycobacterium avium* subsp. *paratuberculosis*; x: recommendation given; N/A: not applicable.

All points of the respective list, including reasons why each measure is particularly important, how it can limit new infections in the herd, and practical aspects for an efficient realization, were discussed with the farmers. In addition, farm-specific weak points favoring MAP transmission observed during the farm visit and practical measures to be implemented on the farm, e.g. building a solid separation instead of a fence made of metal bars between the calf and the cow area, were discussed with the farmers. Farm-specific recommendations were more detailed than the general principles of good hygiene and avoidance of exposure of calves to cow manure given in Table 1, and they were recommended to farmers based on the particular situation of each farm (Table 2).

**Table 2:** Farm-specific recommendations given to the managers of ten dairy and seven beef farms to limit MAP spread on their farms.

Farm-specific recommendations	Dairy	Beef	Total
***Calving hygiene/ calving pen***			
Calving in the calving pen	4	3	7
Emptying of the calving pen after each birth	7	7	14
Cleaning of the calving pen after each birth	7	5	12
Disinfection of the calving pen after each birth	0	5	5
Building of a calving pen	1	1	2
Only one dam and her calf per calving pen	0	2	2
***Separation of newborns from adults / feeding of the calves***			
Separation of the calves from their dam immediately after birth	7	N/A	7
Preventing calves from suckling their dam	4	N/A	4
Use of colostrum from the own dam only	1	N/A	1
Milk replacer instead of milk	10	N/A	10
No feeding of waste milk	7	N/A	7
***Cleaning and disinfection***			
Cleaning the outdoor paddock with high-pressure-wash	1	0	1
Building a concrete floor in the outdoor paddock	0	1	1
Using separate instruments for feed and manure	1	1	2
Avoiding feed contamination by boots or machines	1	2	3
Cleaning and disinfection of the stable(s) once a year	0	1	1
***Separation of the young stock from adults***			
Separation of heifers and dry cows	3	0	3
Separated outdoor paddocks for heifers and cows	1	0	1
Separation of calves / heifers from the calving pen	1	1	2
Separation of the calves from the cows	4	N/A	4
Building a separated area for calves	N/A	2	2
Separated area for newborn calves and their dams	N/A	3	3
Building a separated area for heifers	1	1	2
Providing fresh straw in the calf area	N/A	1	1
Separation of pastures for young stock and adults	2	0	2
***Manure management***			
Preventing access of animals to manure stock	1	1	2
No cow manure on pastures	5	5	10
No use of dried manure in cubicles for heifers	1	0	1
***Total***	**70**	**42**	**112**

MAP: *Mycobacterium avium* subsp. *paratuberculosis*; N/A: not applicable.

Both the list of general recommendations and the list of farm-specific recommendations, including comments and explanation for each proposed measure, were sent in writing to the farmers after the farm visit.

Farm specific recommendations were discussed again with each farm manager individually during the following farm visit. At that time, the farm managers indicated which measures they considered to be implementable on their farms and which ones they actually intended to implement in the coming years. They were asked to start realizing these management changes immediately. In early 2012, all farmers received a written summary of the measures they intended to implement for the subsequent three-year PO.

During the PO, all farms were visited twice yearly, usually once in springtime and once in the fall. The implementation of control measures was evaluated during each visit by use of a short questionnaire and a viewing of the operation. During each visit, the managers were asked specifically, among others, if they had slaughtered animals tested positive or presenting clinical signs suggestive of PTB (chronic diarrhea, weight loss), if new animals had shown clinical signs of PTB, and if any other special occurrences had been observed on the farm. After the PO, all farms were visited for the last time in early 2015. At that point, a detailed questionnaire about relevant changes on the farms during the PO was completed with the farm managers and fecal samples were collected for culture (second testing). A final evaluation of the control measures was performed, and, if some or all measures the farmers had intended to realize had not been implemented, the reasons for this discrepancy were registered.

The project was approved by the Swiss Federal Food Safety and Veterinary Office.

### Detection of *Mycobacterium avium* subsp. *paratuberculosis* (MAP)

All animals aged one year or older had been tested for MAP shedding by fecal culture following the first farm visit in 2011 [6, 20]. After the PO in 2015, fecal samples were collected again (from the rectum with a new glove for each sample) from every animal ≥1 year old. The collected samples were sent to the laboratory by overnight courier and stored at -20°C until further processing. Culture for MAP and identification via F57 PCR were performed with pools of three samples using the same methods as in 2011 [20], except that one culture medium only (egg yolk agar) was used. If a pool was positive for MAP, the three original samples were re-cultured individually. If MAP could not be cultured from the original three samples, the presence of one positive animal per positive pool was assumed for further analyses [6]. Furthermore, the participating farmers had the possibility to have animals with clinical signs suggestive of PTB tested by fecal PCR (real-time PCR [20]) and serum ELISA (IDEXX Paratuberculosis Screening Ab Test, IDEXX Montpellier SAS, France) at no cost during the PO. The samples were taken and submitted to the laboratory by the farm’s private veterinarian.

### Data analyses

For comparison of the implementation of control measures among farms, three levels of implementation (implementation scores) were defined for further analyses: control measures always and completely implemented (score 2, corresponding to two points), control measures implemented but not consistently or not over the entire time of the PO (score 1, one point), and control measures never implemented (score 0, no point). The scores were summed and the percentage of implemented measures was calculated from a possible total (100%) corresponding to two points per recommended measure.

Each farm’s apparent herd prevalence was calculated from the results of all animals ≥2 years old for the time before and after the PO. The young stock were not available for sampling in all farms and were therefore not included in the calculation of herd prevalence. Animals with clinical signs suggestive of PTB tested during the PO were reported separately and were not included in prevalence calculations before and after the PO.

Factors with a potential influence on the farmers’ compliance to implement management recommendations were investigated with multivariable linear regression. These included 1) the knowledge of the farmers about PTB (percentage of correctly answered questions out of 15 questions about symptoms, epidemiology and preventive management practices regarding PTB); 2) the PTB history on the farms (number of years between the first confirmed case of PTB on the farm and the start of the project in 2011); 3) the number of animals tested positive before the project; and 4) the number of animals tested positive at first testing and during the PO. These factors were compared with the percentage of implemented measures (number of effectively implemented measures compared to the number of recommended measures). The distribution of the percentage of implemented measures across the farms were first checked for normality.

Differences in the prevalence of MAP shedding for the period before and after the PO were tested by logistic regression. Potential associations of herd prevalence values with the percentage of implemented control measures out of the recommended measures were explored by use of multivariable linear regression models, the herd was included as random effect in all models. Recommended farm-specific control measures were grouped in five subject areas (calving hygiene/calving pen, separation calves-adults/feeding of the calves, cleaning and disinfection, separation young stock-adults, and manure management; Table 2). The analyses were performed first for all herds and then separately for dairy herds and beef herds.

Statistical analyses were performed in R [21], the level of significance was set at p ≤ 0.05.

## Results

### Farm characteristics

The 17 participating farms were located in nine different cantons of Switzerland, mainly in the western part of the country. Herd size (all animals ≥1 year on the farm) averaged 68 animals in 2011 (73 for dairy herds, 62 for beef herds), and 71 (75 for dairy herds, 65 for beef herds) in 2015. Animals of the Red Holstein, Holstein Friesian, Brown Swiss, Swiss Fleckvieh and Jersey breeds were kept in the dairy farms, and Limousin, Angus and Charolais in beef herds. In six farms (one dairy and five beef farms), a second cattle operation was run in parallel to the main production branch (dairy or beef), such as fattening calves in dairy herds or a separated dairy herd beside the beef herd. Only animals of the main operation were enrolled in the study. Additional workers, beside the owner and his family, were employed on 12 farms. Over all operations, a mean of three and a half persons were involved in the care of the animals in the participating herds.

One of the dairy farms (farm D) underwent a barn fire during the PO (October 2013), which affected its husbandry practices for the rest of the study duration. Some of the animals were also kept in a temporary barn or in another farm in the vicinity until a new barn was available, which had not been realized until the last visit in March 2015. As the manager of this farm continued to apply the discussed recommendations for PTB control after a first period of reorganization, the farm was kept in the project.

### Implementation of control measures

Regarding the main general recommendations given to all herd managers, nine of the 17 farmers (53%) had consistently slaughtered all animals positive in the first testing and during the PO; the remaining eight farmers had sent the majority but not all positive animals to slaughter. The offspring of positive animals was consistently culled by the managers of five farms (29%), and inconsistently or not at all on 6 farms each (35%). Animal purchase was discontinued on 3 farms (18%), reduced to a minimum on 2 farms (12%), while no effort to limit animal movement was made in the majority of the farms (71%).

The number of farm-specific recommendations per farm ranged from three to ten, with a mean of 6.6 proposed measures (seven in dairy, six in beef herds). A large variability was observed in the willingness of the farmers to implement control measures for PTB on their operations. The farmers had intended to implement a mean of 40% of the recommendations (range, 0–100%), corresponding to 53% (37 measures) of all recommendations in dairy farms and 21% (9 measures) in beef farms. Over all farms, herd managers had intended to implement 46 recommendations, 15 of these measures were effectively and completely implemented (33%). The proportion of recommendations that farmers intended to implement across the 17 farms was approximately normally distributed (Shapiro-Wilk normality test, p = 0.38).

Table 3 shows which control measures the farmers indicated they were intending to implement and which ones they actually implemented, and to which degree (implementation score 0–2) over all dairy and beef herds, respectively. Table 4 shows the implementation of general and specific control measures and the within-herd prevalence before and after the PO for each farm individually.

**Table 3:** Recommendations and implementation of general and farm-specific control measures to reduce the spread of paratuberculosis in ten dairy and seven beef farms.

	Dairy farms					Beef farms				
	Recom-mended	Intent	Implementation score			Recom-mended	Intent	Implementation score		
			2	1	0			2	1	0
**Main general recommendations**										
Slaughter of test-positive animals	10	10	6	4	0	7	7	3	4	0
Slaughter of offspring from test-positive animals	10	10	3	2	5	7	7	2	4	1
Avoid/minimize animal purchase	10	10	1	1	8	7	7	2	1	4
Total number of recommended or implemented general control measures	30	30	10	7	13	21	21	7	9	5
Implemention of general control measures in % of the total number of recommendations		100%	33%	23%	43%		100%	33%	43%	24%
**Farm-specific recommendations**										
***Calving hygiene/ calving pen***										
Calving in calving pen	4	4	0	4	0	3	0	0	2	1
Emptying of calving pen after each birth	7	3	0	4	3	7	3	0	4	3
Cleaning of calving pen after each birth	7	2	0	3	4	5	0	0	2	3
Disinfection of calving pen after each birth	0	N/A^2^	N/A^2^	N/A^2^	N/A^2^	5	1	0	1	4
Installation of a calving pen	1	1	1	0	0	1	0	0	0	1
Only one dam and her calf per calving pen	0	N/A^2^	N/A^2^	N/A^2^	N/A^2^	2	0	0	2	0
***Separation calves/ feeding calves***										
Separation of calves from dam directly after birth	7	6	1	6	0	N/A^3^				
Prevent calves suckling their dam	4	1	0	4	0	N/A^3^				
Milk replacer instead of milk	10	2	2	0	8	N/A^3^				
No feeding of waste milk	7	3	1	5	1	N/A^3^				
Use of colostrum from dam only	1	1	1	0	0	N/A^3^				
***Separation young stock from adults***										
Separation of heifers and dry cows	3	2	0	3	0	0	N/A^2^	N/A^2^	N/A^2^	N/A^2^
Separated farmyard for heifers and cows	1	0	0	0	1	0	N/A^2^	N/A^2^	N/A^2^	N/A^2^
Separation of calves / heifers from calving pen	1	0	0	0	1	1	1	1	0	0
Separation of calves from cows	4	4	2	2	0	N/A^3^				
Separated area for calves	N/A^1^					2	0	0	0	2
Separated area for new born calves and their dams	N/A^1^					3	0	0	2	1
Separated area for heifers	1	1	1	0	0	1	0	0	0	1
Fresh straw in calves’ area	N/A^1^					1	0	0	1	0
Separated pastures for young stock and adults	2	1	0	1	1	0	N/A^2^	N/A^2^	N/A^2^	N/A^2^
***Cleaning and disinfection***										
Cleaning of the farmyard by high-pressure-wash	1	1	1	0	0	0	N/A^2^	N/A^2^	N/A^2^	N/A^2^
Ground fixation of the farmyard	0	N/A^2^	N/A^2^	N/A^2^	N/A^2^	1	0	0	0	1
Separated machines for feed and manure	1	0	0	0	1	1	0	0	1	0
Avoid contamination of feed by boots or machines	1	1	1	0	0	2	1	0	2	0
Cleaning and disinfection of stable once a year	0	N/A^2^	N/A^2^	N/A^2^	N/A^2^	1	0	0	0	1
***Manure management***										
Preventing access to manure stock	1	1	1	0	0	1	1	1	0	0
No cow manure on pastures	5	2	0	1	4	5	1	0	3	2
No use of dried manure in cubicles for heifers	1	1	1	0	0	0	N/A^2^	N/A^2^	N/A^2^	N/A^2^
Total number of recommended or implemented farm-specific measures	70	37	13	33	24	42	9	2	20	20
Implemention of farm-specific control measures in % of the total number of recommendations		53%	19%	47%	34%		21%	5%	48%	48%

Intent = measures the farmers had indicated they intended to realize; implementation scores for control measures: 2 = measure always and completely implemented, 1 = measure not always or not completely implemented, 0 = measure never implemented; N/A^1^ = not applicable for dairy farms; N/A^2^ = not applicable because the measure was never recommended; N/A^3^ = not applicable for beef farms.

**Table 4:** General and farm-specific control measures to reduce the spread of paratuberculosis implemented in comparison with within-herd prevalence of MAP shedding at the beginning and at the end of the observation period for each farm individually.

		2015 herd size (>1 year)	2011 prevalence (≥2 years)	2015 prevalence (≥2 years)	Slaughter of positive animals during the project (implementation score)	Slaughter of offspring of positive animals (implementation score)	Avoid/minimize animal purchase (implementation score)	Number of farm-specific recommendations (in points)	Number of farm-specific recommendations the farmers intended to implement (in points)	Total implementation score (for farm-specific recommendations)	% of recommended farm-specific measures effectively implemented
Farm	Herd type										
A	Dairy	75	5.3%	5.6%	2	1	0	8	8	5	63%
B	Dairy	37	3.6%	3.3%	2	0	0	10	4	5	50%
C	Dairy	108	7.3%	6.0%	2	2	0	14	2	2	14%
D	Dairy	142	12.9%	7.0%	1	0	1	16	10	7	44%
E	Dairy	64	1.9%	3.5%	2	2	0	12	6	5	42%
F	Dairy	49	5.1%	5.0%	2	2	0	18	14	10	56%
G	Dairy	87	6.5%	3.2%	1	1	0	20	14	11	55%
H	Dairy	51	6.3%	0.0%	1	0	2	12	2	2	17%
I	Dairy	60	4.5%	2.3%	1	0	0	16	10	6	38%
J	Dairy	78	1.2%	0.0%	2	0	0	14	4	6	43%
K	Beef	39	7.1%	9.4%	1	1	0	8	6	5	63%
L	Beef	66	7.8%	10.6%	1	1	2	10	2	1	10%
M	Beef	60	1.9%	8.3%	1	1	1	6	4	2	33%
N	Beef	81	3.6%	8.1%	1	0	2	18	0	0	0%
O	Beef	123	8.8%	1.2%	2	2	0	14	0	4	29%
P	Beef	39	12.9%	0.0%	2	2	0	16	0	5	31%
Q	Beef	47	2.5%	5.0%	2	1	0	12	6	7	58%
**Mean**			**5.8%**	**4.6%**	**1.5**	**0.9**	**0.5**	**13.2**	**5.4**	**4.9**	**38%**

Implementation scores for control measures: 2 = measure always and completely implemented, 1 = measure not always or not completely implemented, 0 = measure never implemented.

The most common reasons mentioned by the participating farmers for not implementing control measures were lack of time and difficulties to get husbandry processes reorganized. Furthermore, several farmers did not observe an impact of the disease in their herds, as there were no animals in the clinical stage of PTB, and they were therefore not motivated to put efforts into the implementation of control measures.

In 13 of the 17 farms (i.e. in all herds except for 2 beef and 1 dairy farm; in the last beef farm no measures were implemented at all), some of the recommended measures were implemented at the beginning of the PO and were later partially or completely discontinued. These measures are shown in Table 5.

**Table 5:** Number of farms where the implementation of control measures to reduce the spread of paratuberculosis was partially or completely discontinued in the course of the three-year period of observation.

Control measures with discontinued implementation	Total	Dairy	Beef
	*n*	*n*	*n*
No feeding of waste milk	5	5	N/A
Separation of tools used for adults and young stock	5	4	1
Separated pastures and/or farmyard for cows and young stock	4	4	0
No cow manure on pastures	4	3	1
Emptying of calving pen after each birth	3	1	2
Separation of cows (including dry cows) and young stock	3	3	0
Avoiding contamination of feed by boots or machines	1	0	1
Avoiding using calving pen as a sick pen	1	0	1

n = number of farms; N/A: not applicable.

The duration of the PTB history in the participating farms varied from 0 to 12 years between the first diagnosed case and the start of the project. The number of confirmed PTB cases per farm before the study begin showed an accordingly wide range (1–21; mean = 4.1). The number of PTB cases before the start of the project, the number of positive animals at the time of first testing in 2011, and the number of animals tested positive during the PO were not statistically significantly associated with the % of implemented control measures (p = 0.35, p = 0.37, p = 0.28 respectively). Likewise, no statistically significant association was observed between the farmers’ compliance (i.e. the percentage of recommended control measures that were implemented) and the farmers’ knowledge about PTB (p = 0.304) or the duration in years of PTB presence in the operation (p = 0.96).

### Fecal culture results and herd prevalence of MAP shedding

During the first testing in 2011, a total number of 1164 cattle had been tested by fecal culture, of which 964 animals were ≥2 years old. By the time of the second testing in 2015, 1206 animals were enrolled, and 993 animals out of these were ≥2 years. At the beginning of the project, the mean prevalence of MAP shedding had been 5.8% for animals ≥2 years (5.5% in dairy herds, 6.4% in beef herds). On two farms (one dairy herd and one beef herd), the within-herd prevalence for animals ≥2 years had been above 10% with 12.9% for both operations [6]. After the PO, the prevalence of MAP shedding averaged 4.6% for animals ≥2 years (3.6% in dairy herds, 6.1% in beef herds). Results of the second testing period revealed only one beef farm with a prevalence over 10% (10.6%), which was not the same farm as the one with a prevalence of 12.9% in 2011 (Table 4). Of the 993 cattle over 2 years of age that were tested in 2015, 286 had already been tested (negative) in 2011; 13 of these animals (4.5%) were positive in 2015. Of the remaining 707 animals tested in 2015 that had not been tested in 2011, 591 had been born on the farms and 115 had been purchased (33 prior to 2011 and 82 afterwards); the origin of one animal could not be determined with certainty. Thirteen of the 591 animals over 2 years of age born on the farms were tested positive in 2015 (2.2%), while three of the 115 purchased animals (2.6%, all added to the herds after 2011) were positive. No statistically significant difference in prevalence for the time before and after the PO was observed (p = 0.765). In three farms (two dairy farms and one beef farm), no animals were tested positive in the frame of the second testing.

Of the 1206 animals tested in 2015, 588 were four years or older. Out of these 588 animals, 365 (62%) had already been on the farm in 2011 and had been tested for MAP shedding during the first testing. Regarding the individuals tested positive in 2015, 22 out of the 41 cows were older than four years, 17 of them (77%) were already listed in 2011 and had been tested negative. Twenty of these 22 cows had been born on the farm and 2 had been purchased, of which one had already been present on the farm at first testing and had been negative. The other purchased animal had not been on the farm at the time of first sampling. The remaining 19 animals had not been available for sampling at first testing because they were on a summer pasture or in a heifer rearing facility.

Fourteen of the 17 farm managers had animals with clinical signs suggestive of PTB tested during the PO. Samples from 31 suspect animals were submitted for testing between 2012 and 2015, and infection with MAP was confirmed by PCR, ELISA, or both for 16 animals. Most animals (15 of 31) were tested during the first year of the PO, of which four (26.6%) were positive. During the following years (2013–2015), three to seven samples of animals with suspect clinical signs were submitted per year. The percentage of animals tested positive was 57–100% for the years 2013–2015.

Culling test-positive animals, with or without their direct offspring, was not significantly (p>0.05) associated with the change of prevalence before and after the PO. In contrast, separating the young stock from adult cattle was significantly (p = 0.02) associated with a higher prevalence of MAP shedding in 2015. Otherwise, no significant associations were found between the implementation rate of control measures, overall or within the five defined subject areas of individual measures, and the change in prevalence of MAP shedding.

## Discussion

The present investigation conducted in ten dairy and seven beef herds infected with PTB in Switzerland revealed a low willingness of herd managers to invest time and efforts into non-mandatory measures to control the disease in the absence of financial or social incentives. The recommended measures to control PTB were only partially implemented and the participating farmers’ concerns about the disease and its economic impact were limited.

The implementation of the three main general recommendations given to all farmers, i.e. culling of test-positive animals, culling of their last offspring, and avoidance or at least severe limitation of animal purchase, was rather low, although all farmers had expressed the intention to realize them. Large differences were observed, with culling of positive animals implemented in all farms (53% completely and 47% partially), while this was the case only in 29% und 35%, respectively, for the offspring of infected animals (these were not culled in the remaining 35% of the farms). Compliance was lowest for restrictions on animal purchase, this recommendation was ignored in 71% of the farms. Cattle trade and animal movements (e.g. to and from alpine pastures in the summer, or to specialized heifer rearing operations as weaned calves to return as pregnant heifers for calving) are very intensive in Switzerland [22, 23], and the awareness of cattle farmers about the importance of biosecurity measures is low. Indeed, most of the farmers (14 out of 17) purchased animals during the PO despite the specific recommendation given to all of them at the beginning of the study to avoid animal purchase or, if purchase was unavoidable (e.g. for heifer replacement in dairy herds or bull replacement in beef herds), to keep it at the lowest possible level and to buy from farms with no known history of paratuberculosis. This last point was implemented in one case only. Some of the farmers purchased large numbers of animals over the PO (corresponding to 17% of the herd size on average over all herds); the bull(s) in four of seven beef herds were replaced regularly.

Over all farms, the percentage of implemented farm-specific measures to reduce the within-herd MAP transmission was low, with 13% of the recommended measures completely implemented, 47% partially, and 39% not at all. The average percentage of implemented farm-specific control measures (38%) was similar to that observed among Canadian dairy farmers enrolled in a JD control program, where the reported mean percentage was 33% [12]. The intention to implement control measures was higher in dairy than in beef farms, with 53% and 21%, respectively. Approximately half of the recommended measures were partially realized in both farm types (47% and 48%), but less were completely implemented (5% vs. 19%) and more were not realized at all (48% vs. 34%) in beef farms compared to dairy farms. Not all measures the farmers had indicated to be willing to implement were realized, but also several measures were implemented, at least partially, by farmers who had expressed no intention to realize that specific measure at the beginning of the PO. Based on the observations made during the first farm visit, the highest number of farm-specific recommendations were given to improve hygiene in the calving pen and minimize exposition of the newborn calves to MAP immediately after birth. Recommended measures to improve this area were poorly implemented, at best partially, as only one recommended measure (installation of a separate calving pen) was consequently implemented (score 2) in one dairy farm. Recommendations regarding calf management prior to weaning in dairy farms were mostly implemented partially, but only few were consistently realized. Interestingly, a measure that may appear easy to implement, feeding milk replacer instead of fresh milk, was only realized in two farms, possibly because of the visible costs associated with the purchase of milk replacer. Management changes aimed at limiting contact between young stock and adult cows were variably implemented, mostly only in part. Cleaning routines were improved in a few farms, in particular by improving prevention of feed contamination with boots or machines. The recommendation not to use cow manure to fertilize pastures was poorly implemented, especially in dairy farms as it was recommended in five farms and only implemented partially in one (vs. partial implementation in three of five beef farms). This poor implementation may also have been due to the costs of purchasing fertilizer to replace manure. Also, several measures aimed mostly at reducing the exposure of the young stock to MAP were realized at first and then abandoned in the course of the PO.

The reasons given for the limited compliance of farmers to improve their management practices differed in each herd, however lack of time and the absence of a visible impact of the disease on the success of the operation were the reasons mentioned most often by the farmers. In the light of our knowledge of the farms’ structure (organization, social factors and financial situation), lack of time as the primary factor preventing the implementation of control measures seems rather unlikely in most farms; only few of them were operated by a farmer alone, and, in our appreciation, the economic situation of the farm (e.g. stress due to low milk prices) or personal circumstances (e.g. barn fire or family matters) were likely more often important components of the perceived lack of time.

In general, the farmers’ knowledge about the disease itself and about the most common risk factors for transmission among animals is an important point to consider in the frame of control programs. In our study, the farmers’ knowledge about PTB had been assessed at the study start [6], a statistically significant association with the consecutive rate of control measure implementation was not observed. Understanding the infection pathways and knowing the economic consequences of the disease has been shown to influence farmers’ compliance in control programs, as dairy farmers who improved their level of knowledge about PTB in the frame of a control program in Ontario did more often implement on-farm changes [24]. However, others reported that PTB was often not considered as a big problem for the herd or an important topic to discuss with their veterinarian by farmers in Alberta, i.e. in another region of the same country [25]. In another recent study in Canada [26], two main barriers to the implementation of control measures against PTB in dairy herds were identified, i.e. practical limitations in the implementation of the recommended measures (time, money and infrastructural constraints), and producer mindset (perceived importance of JD control, e.g. regarding the zoonotic potential of MAP in relation to Crohn’s disease in humans, and practicability of control measures). Keeping a closed herd or buying from low-risk herds, the general recommendation with the poorest implementation in our study, was mentioned by the producers as unrealistic and too difficult to realize in that study as well [26]. In contrast, culling test-positive animals, the most consequently implemented measure in the present study, was also found to be the measure with the highest compliance in Canada [12]. Thus, although comparable data are not available from European countries, the perception of PTB and the motivation of dairy farmers toward control measures appear to be similar across countries and continents. Similar perceptions and motivations have been described for beef farmers in North America as well [27, 28].

The large number of measures that can potentially be recommended on an infected farm to control the many MAP transmission pathways may lead to excessive demand for the farmers. In the present study, a mean number of 6.6 specific measures was recommended per farm. This rather high number of recommendations must be put into perspective with the fact that the farmers themselves could define which measures they intended to implement or not. An alternative procedure for recommendations under practice conditions would be to define a smaller number of management changes for a shorter period of time. In a Canadian study in dairy farms, the herd veterinarians recommended different measures and then defined three management changes to be implemented every year with the farmers [29]. An additional advantage of this procedure is that the discussion with the veterinarian helps the farmers to choose the most relevant management changes, and not just the easiest or cheapest ones. Among Canadian dairy farmers, easy-to-implement management changes, those with a visible effect, or management improvements associated with low costs were the ones that were most often chosen by the producers [12]. A similar pattern was observed in our study, as measures requiring important changes in the herd routine, e.g. better hygiene at calving, improved pasture management or control of animal movement, were poorly implemented, while culling of test-positive animals, that can be easily related to an immediate benefit for the farm, was realized at least partially in all farms. In any case, a detailed assessment of the farm and its routine husbandry practices is mandatory to identify the most important risk factors for transmission of PTB on a particular farm [4, 15]. Furthermore, a clear definition of the goals of a control program as well as regular farm visits and constant support of the farmers increases their compliance to implement control measures [15]. Involvement of a trusted professional with good knowledge of the farm’s circumstances, i.e. of the farm veterinarian, helps the farm managers to keep their motivation and to implement management improvements [25, 30]. In the frame of the research project reported here, the biannual farm visits were realized by the study team, i.e. by veterinarians from the university; the private veterinarians were informed about the project and all available results, e.g. if a suspect animal was tested during the PO, but they were not directly involved in the implementation of control measures. Should an official control program for PTB be launched in the future, direct participation of the herd veterinarians to support the farmers in the implementation of the program would be mandatory.

Economic considerations can also be a motivator for implementing management changes, depending on the awareness of the farm manager about the economic impact of the disease. Several authors have reported that the losses associated with PTB are often underestimated by farmers [12, 28, 30]. In small herds as in our study, the number of clinical cases on the farm and, therefore, the directly visible losses due to PTB may be relatively low. Moreover, in comparison to bigger herds, the costs for modifications of existing buildings, such as spatial reorganization for an additional calving box, are proportionally higher for small farms. Since participation in the project and compliance with recommended control measures was completely voluntary in this project, there was no economic motivation to implement control measures for the participating farmers as they did not face any restrictions, e.g. regarding animal sale or purchase, related to their positive infection status. The Swiss federal law about epizootic diseases has been modified in 2015: a temporary ban on milk sales in dairy herds and animal movement restrictions are ordered if an animal is diagnosed with JD, immediate culling of the positive animal is mandatory and an official veterinarian inspects the herd for other suspect cattle. These measures are lifted after all suspect animals have been culled or tested negative. Beside financial aspects, involvement of an official veterinarian signals the significance of the disease to farm managers and may increase their awareness about the need for a better control of PTB in the future.

A stable within-herd prevalence of MAP shedding was observed in the 17 participating farms despite sparse implementation of the proposed management improvements. An association between the farmers’ compliance and potential influencing factors such as their knowledge about PTB or the history of JD on the farm was not observed. Furthermore, neither a particular group of farm-specific control measures nor the total percentage of implemented management changes was significantly associated with within-herd prevalence of MAP shedding after the PO. These results must be interpreted with caution because of the limited PO of 3 years (vs. the long incubation time of PTB). However, given the low implementation rate of control measures observed in this study, significant changes in prevalence would likely not have become evident even after a longer PO. Beside the within-herd prevalence, production data collected on the farms during the PO (milk yield or daily weight gain in dairy and beef farms, respectively; data not shown) suggested a stable situation in the participating farms over the three years of the study. The only statistically significant (positive) association was seen between the separation of young stock from adult cattle and a higher prevalence of MAP shedding after than before the PO; this observation cannot be explained reasonably, except by the fact that the managers of herds with higher prevalence, more losses due to the disease, or both, may rather have implemented this central control measure to reduce exposure of young animals. The focus of this investigation was on compliance with recommendations to control PTB on infected farms, not on the prevalence of fecal MAP shedding as cultures could be performed only once at the beginning and once at the end of the PO due to practical constraints. Therefore, these results may certainly not be interpreted as a proof of lack of efficacy for control measures to prevent MAP spread, given the limited number of participating farms and the low implementation rate of control measures.

Since the farm managers involved in the study had agreed to participate voluntarily in a research project extending over several years, an above-average motivation and interest for the disease can be assumed. Nonetheless, these farmers only implemented some of the recommended control measures, which likely indicates that the perceived losses associated with PTB were not considered sufficient to justify profound changes in herd management strategies.

## Conclusions

The managers of infected Swiss dairy and beef herds were reluctant to implement control measures to minimize MAP spread in the frame of their voluntary participation in a research project conducted over a period of three years. Except for culling test-positive animals, implementation rates were low for most proposed measures, and some of those that were implemented at the beginning of the PO were discontinued in the course of the study. The farmers mostly implemented cheap and easy measures, while changes impacting farm structure or their routines more deeply, e.g. improving hygiene in the calving area or minimizing animal purchase, were rarely realized. The farmers did not observe relevant economic losses due to PTB in their herds and therefore did not expect an immediate reward for expenditures in time, efforts and money. These observations suggest that incentives for a consequent implementation of control measures should be included in future programs to increase the compliance of the managers of herds infected with PTB and thus improve the chances of successfully controlling the disease.

## Supporting information

S1 Checklist STROBE Statement—Checklist of items that should be included in reports of *cohort studies*. (PDF)
